# Gastric Negative Pressure Suction Method Reduces the Incidence of PONV after Orthognathic Surgery

**DOI:** 10.3389/fsurg.2022.882726

**Published:** 2022-05-20

**Authors:** Jia Wang, Zhenzhen Zhang

**Affiliations:** ^1^ R.N., School and Hospital of Stomatology, China Medical University, Liaoning Provincial Key Laboratory of Oral Diseases, Shenyang, China; ^2^ Resident, School and Hospital of Stomatology, China Medical University, Liaoning Provincial Key Laboratory of Oral Diseases, Shenyang, China

**Keywords:** gastric negative pressure suction, orthognathic surgery, PONV, operation time, retrospective study

## Abstract

**Objective:**

To investigate the effect of gastric negative pressure suction on the incidence of postoperative nausea and vomiting (PONV) in patients undergoing orthognathic surgery.

**Methods:**

A retrospective study of 772 patients who underwent orthognathic surgery from October 2016 to January 2021 was performed, excluding possible confounding factors. The patients were divided into a negative gastric suction group (group 1) and a group without gastric suction (group 2), according to whether gastric suction was used after general anaesthesia. There were 386 patients in each group. The incidence of PONV was compared between the two groups.

**Results:**

The incidence of PONV was 29% in the negative gastric suction group and 58.8% in the non-gastric suction group. The incidence of PONV in the gastric negative pressure suction group was significantly lower than that in the non-gastric negative pressure suction group, and the difference was statistically significant (*p *< 0.05).

**Conclusion:**

By reducing the risk of perioperative bleeding in orthognathic surgery, gastric negative pressure aspiration can reduce the incidence and operation time of PONV after orthognathic surgery.

## Introduction

Postoperative nausea and vomiting (PONV) have always been a worrying problem after general anaesthesia. The incidence is estimated at 30% in the general surgical population and up to 80% in high-risk cohorts (1). In orthognathic surgery, the proportion is 40% (1–3). Patients undergoing orthognathic surgery were 6.75 times as likely to experience vomiting than those who had other types of surgery (4). PONV can cause bleeding, delayed healing, and wound infection. A painful experience and even accidental inhalation of vomit may endanger the patient’s life.

Gastrointestinal decompression is the use of negative pressure suction, also known as gastric negative pressure suction, which refers to the suction of gastrointestinal contents to reduce internal pressure. A gastrointestinal decompressor or other instrument is used to suck out food, gas, secretions, exudates, etc., from the gastrointestinal tract to relieve the adverse consequences caused by increased internal pressure. It is mostly used for patients before gastrointestinal surgery to facilitate surgery. It is also used for patients after gastrointestinal and abdominal surgery to prevent negatively affecting the results of surgery. In patients with gastrointestinal tumours or other inoperable conditions, the gastrointestinal tract accumulates too much, so the treatment relieves symptoms and pain.

Studies have shown that PONV is associated with many factors, such as anaesthetic drugs, opioids, age, sex, body mass index (BMI), surgical site, and body habits (5). However, there is no mention of whether the gastric negative pressure suction method to reduce intragastric blood stimulation after orthognathic surgery can reduce the incidence of PONV.

Through postoperative observation and communication with patients to reduce the incidence of postoperative PONV, we found that intragastric blood stimulation in patients with orthognathic surgery may also lead to PONV (6, 7), especially within 24 h of the operation. Therefore, this study mainly discusses the relationship between gastric negative pressure suction and the incidence of PONV after orthognathic surgery.

## Materials and Methods

### Clinical Data

This study followed the Declaration of Helsinki and was approved by the hospital ethics committee. This retrospective study included the clinical data of patients who underwent orthognathic surgery in our hospital from October 2016 to January 2021. Inclusion criteria for this study: (a) patients over 18 years who underwent maxillary or mandibular orthognathic surgery; (b) patients with postoperative PONV (including nausea, vomiting, or retching); and (c) nursing records showing that PONV occurred within 24 h in PACU and ICU. Exclusion criteria: patients with gastrointestinal problems, dizziness, migraine, and pharyngitis were excluded from this study. Finally, 772 patients were selected from 802 patients.

### Method

Both groups used intravenous inhalation combined anaesthesia. The anaesthetics used included sevoflurane, oxygen, sufentanil, fentanyl, and propofol. There was no difference in medication between the two groups. The surgical site was divided into three categories: (1) maxillary procedures (maxillary osteotomies); (2) mandibular procedures (mandibular sagittal osteotomies, distraction osteogenesis, genioplasty); and (3) bi-jaw procedures (maxillary and mandibular osteotomies).

On the doctor’s decision, all patients returned to PACU after orthodontic surgery without intermaxillary fixation. No nasogastric tube was used in any patients to reduce the irritation of the stomach tube to the patient’s throat.

Group 1: After the orthognathic surgery, the patients were returned to PACU. Patients under general anaesthesia were not fully awake. Doctors and nurses administered further anaesthesia to facilitate the study. We inserted tubes on one side of the patient’s nasal cavity to attract nasal mucus and blood, then inserted tubes into the stomach to attract the stomach blood, gas, and gastric juices. An anaesthesiologist gave patients anti-nausea drugs (Tropisetron Hydrochloride Injection, 5 mg IV) at the end of the operation and, based on the clinical situation, gave analgesic and sedative drugs. Group 2: gastric negative pressure suction was not used after the operation. The changes in operation time and postoperative PONV incidence were compared between the two groups.

In previous studies, increased BMI, gender differences, maxillary surgery, and prolonged operation time all led to an increase in the incidence of postoperative PONV. The incidence of PONV in women is higher than that in men, and increased blood loss results in increased blood swallowing during maxillary surgery. Therefore, we compared whether there were statistically significant differences between the two groups in terms of gender, operation time, operation methods, BMI, and so on (3, 4, 5, 8). The results showed no statistical difference between the two groups (see Table 1).

**Table 1:** Patient demographics of Gastric negative pressure suction method group and no Gastric negative pressure suction method group.

Sub group		Group 1 (*n* = 386) Gastric negative pressure suction method group	Group 2 (*n *= 386) No gastric negative pressure suction method group	X^2^	*p* value
Sex	Male	137	158		
	Female	249	228	2.419	0.12
BMI ≥ 25 (kg/m2)		129	101	0.751	0.386
Usage of opioids intraoperatively	Sufentanil Citrate only Fentanyl citrate only	301 85	322 64	3.668	0.055
Modus operandi	Maxillary procedures	1	3		
	Mandibular procedures	136	115		
	Bi-jaw procedures	249	268	2.114	0.146
Duration of surgery (min)	<180 min	112	89		
	>180 min	274	297	3.558	0.059

*Note: Patient demographics of Gastric negative pressure suction method group and no Gastric negative pressure suction method group. Comparison of gender, BMI, duration in surgery (min) and modus operandi of between the two groups. Whether there are statistical differences. The results showed that there was no statistical difference between the two groups*.

The evaluation standard adopts the PONV evaluation method recommended by the World Health Organization. Grade 0 means that the patient does not have nausea and vomiting. Grade I means that the patient has slight nausea, abdominal discomfort, and no vomiting. Grade II means that the patient has moderate nausea and obvious vomiting. Grade III means that the patient has severe nausea and vomiting with vomiting of gastric contents, and this was difficult to control with drugs. We observed and asked for patients’ subjective symptoms in real-time, recorded the severity of postoperative nausea and vomiting, strictly implemented the evaluation criteria, and took the patient’s feelings when lying still as criteria. We compared the first exhaust time and defecation time after the operation between the two groups. We compared the number of cases requiring the postoperative rescue antiemetic drug metoclopramide injections between the two groups.

### Statistical Analysis

SPSS 26.0 statistical software was used to sort and analyse the data, and the results were statistically analysed by Chi-square tests of nonparametric variables. The results were considered significant at the critical level of 5% (*p* < 0.05).

## Results

Patients were divided into a gastric suction group (group 1) and a non-gastric suction group (group 2) according to whether gastric suction was used after general anaesthesia, with 386 patients in each group. Group 1 consisted of 137 males and 249 females. Group 2 consisted of 158 males and 228 females. There were no significant differences between the two groups. The average body fat of group 1 was 129, and that of group 2 was 101. In the first group, the operation time was less than 180 min in 112 cases and more than 180 min in 274 cases. In group 2, the operation time was less than 180 min in 89 cases and more than 180 min in 297 cases. The operation time of the first group was shorter than that of the second group, as shown in Table 1.

**Figure 1:**
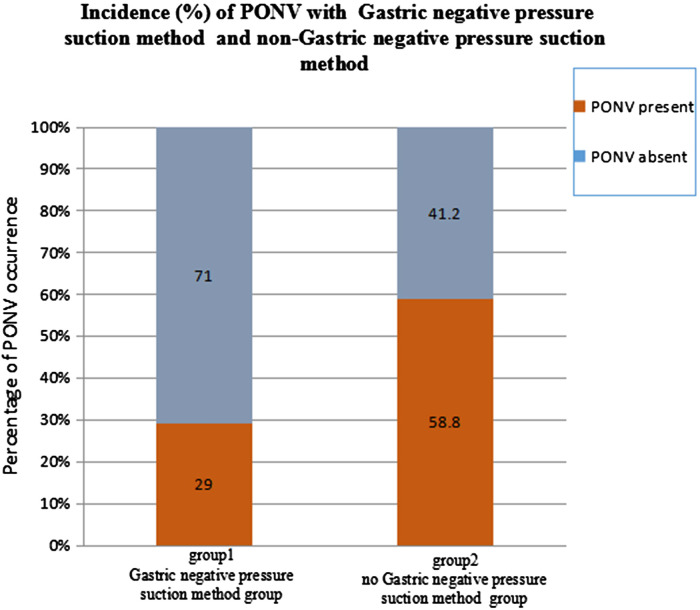
The incidence of PONV in patients undergoing the Gastric negative pressure suction method after orthognathic general anaesthesia was 29.8% lower than that in patients without the Gastric negative pressure suction method.

The incidence of PONV was 29% in group 1 and 58.8% in group 2. The incidence of PONV in group 1 was significantly lower than in group 2 (*p *< 0.05), as shown in Table 2 and Figure 1.

**Table 2:** PONV results for the Gastric negative pressure suction method and no g Gastric negative pressure suction method.

Sub group	Group 1 (*n *= 386)	Group 2 (*n* = 386)	X^2^	*p* value
	Gastric negative pressure suction method group	No gastric negative pressure suction method group		
PONV present	112 (29.0%)	227 (58.8%)	69.555	0.000
PONV absent	274 (71.0%)	159 (41.2%)		

## Discussion

After oral and maxillofacial surgery, patients are prone to complications such as nausea, vomiting, pain, infection, and scars. The most common postoperative complications are the incidence of nausea and vomiting at about 40%, which seriously affects the prognosis quality of patients and the overall curative effects of surgical treatment (9).

The occurrence of PONV includes the comprehensive results of various factors, including surgical, patient, and anaesthesia factors. Since 2015, we have been making continuous improvements in reducing the incidence of PONV after orthognathic surgery, such as preoperative psychological counselling, shortening the duration of general anaesthesia, applying antiemetic drugs in time after surgery, reducing the application of anaesthetic drugs that can easily cause PONV in patients, and reducing intraoperative bleeding.

In addition, in previous studies, gastric tubes also caused postoperative PONV by stimulating the glossopharyngeal nerve. Indwelling gastric tubes caused chronic stimulation of the pharynx and larynx, and PONV. After orthognathic general anaesthesia, doctors would decide whether to use gastric tubes according to the patient’s condition. However, it was found that patients using gastric tubes after orthognathic surgery had poor tolerance to gastric tube stimulation. This increased the incidence of PONV and delayed postoperative recovery (10, 11). Since 2015, gastric tubes have no longer been used in patients receiving general anaesthesia while undergoing orthognathic surgery in our hospital, but oral care has been increased to reduce the possibility of postoperative PONV and intraoral wound infection.

Previous studies have also proposed whether a pharyngeal pack is performed to absorb blood and irrigation fluids and provide a physical barrier against aspiration of surgical debris into the aerodigestive passages and oesophagus, and reduce the incidence of postoperative PONV, such as the application of pharyngeal packs in nasal surgery and minor oral surgery. However, through analysis and research, it is concluded that a pharyngeal pack has no significant effect on reducing the occurrence of PONV, and some studies suggest that a pharyngeal pack should not be used during surgery. In this study, some patients also received a pharyngeal pack during their operation, but because previous studies showed that a pharyngeal pack had no significance for the incidence of postoperative PONV, these patients were not included in this study (12–15).

In this study, we also excluded patients with the previous PONV, vertigo, migraine, and pharyngitis from this study to reduce confounding factors (1, 5–8). In order to reduce the possibility of perioperative bleeding, patients with hypertension before an operation should take active treatment to control their blood pressure to within the normal range. Controlled hypotension during anaesthesia in Lefort I osteotomy can be regarded as a safe method for reducing intraoperative blood loss and, in turn, improving the surgical field (16).

There are limitations to our study. A team of 11 anaesthesiologists was used, and each anaesthesiologist was responsible for one orthognathic surgery patient. Therefore, there are differences in anaesthesia induction, medication, and medication methods. At the same time, there were three teams that could perform orthognathic surgery, and at least two patients underwent orthognathic surgery every day. The intraoperative and postoperative blood loss of each team was also different. This study is a retrospective study, so there may be bias. It is hoped that a prospective study can be carried out in the future.

## Conclusion

In this study, we found intraoperative and postoperative blood swallowing, intraoperative flushing fluid into the stomach, and gastric gas during anaesthesia are risk factors for postoperative PONV. To reduce the risk of bleeding during the perioperative period in patients undergoing orthognathic surgery, the gastric negative pressure suction method after orthognathic surgery can reduce stimulation to the stomach and gastric pressure, thus reducing the incidence of postoperative PONV. During peri-orthognathic surgery, through psychological intervention, comprehensive observation, and timely and accurate records, nurses can take the correct gastric negative pressure suction method. This is of great significance in reducing the incidence of PONV in patients after surgery.

## Data Availability

The original contributions presented in the study are included in the article/Supplementary Material, further inquiries can be directed to the corresponding author/s.
